# A Simulation-Based Comparison of Covariate Adjustment Methods for the Analysis of Randomized Controlled Trials

**DOI:** 10.3390/ijerph13040414

**Published:** 2016-04-11

**Authors:** Pierre Chaussé, Jin Liu, George Luta

**Affiliations:** 1 Department of Economics, University of Waterloo, Hagey Hall of Humanities, Waterloo, ON N2L 3G1, Canada; pierre.chausse@uwaterloo.ca; 2 Department of Biostatistics, Bioinformatics and Biomathematics, Georgetown University Medical Center, 4000 Reservoir Road NW, Washington, DC 20057, USA; jl1830@georgetown.edu

**Keywords:** randomized controlled trials, ANCOVA, empirical likelihood, exponential tilting, continuous updated estimator, generalized empirical likelihood

## Abstract

Covariate adjustment methods are frequently used when baseline covariate information is available for randomized controlled trials. Using a simulation study, we compared the analysis of covariance (ANCOVA) with three nonparametric covariate adjustment methods with respect to point and interval estimation for the difference between means. The three alternative methods were based on important members of the generalized empirical likelihood (GEL) family, specifically on the empirical likelihood (EL) method, the exponential tilting (ET) method, and the continuous updated estimator (CUE) method. Two criteria were considered for the comparison of the four statistical methods: the root mean squared error and the empirical coverage of the nominal 95% confidence intervals for the difference between means. Based on the results of the simulation study, for sensitivity analysis purposes, we recommend the use of ANCOVA (with robust standard errors when heteroscedasticity is present) together with the CUE-based covariate adjustment method.

## 1. Introduction

When baseline covariate information is available for randomized controlled trials in the areas of environmental research and public health, statistical methods that perform covariate adjustment are usually employed. There are two main reasons to use covariate adjustment methods for the statistical analysis of randomized experiments: one is variance reduction for the estimators for the parameters of interest, which will lead to narrower confidence intervals and more powerful statistical tests; the other is to achieve the equivalence of the treatment groups that is expected as a consequence of randomization [1]. We note that under Neyman’s causal model for randomization inference, the use of ordinary least squares regression covariate adjustment may increase the asymptotic variance in some cases [2]. This issue can be addressed by the inclusion of treatment by covariate interactions, or by the use of robust standard error estimators [3].

An example of a randomized controlled trial is the randomized study from Lanphear *et al.* (2000) [4] that investigated the long-term effect of dust control on blood lead concentrations. The participants were 275 children from Rochester, New York, who were randomized (together with their families) at six months of age to an intervention group (that received cleaning equipment and up to eight visits by a trained lead hazard control advisor) or to a control group. The intervention was terminated when the children were 24-months of age. The outcome for this experimental study was the natural log transformed blood lead concentration at the 48-month follow-up, while the natural log transformed blood lead concentration at the six-month baseline may be used as a covariate.

The analysis of randomized controlled trials, like the one described above, is usually performed using the classic analysis of covariance (ANCOVA). ANCOVA is a method that combines features of the analysis of variance (ANOVA) and the linear regression [5]. It is a popular parametric method used to compare the means of the outcome variables for different treatment groups while controlling for the covariates. ANCOVA may involve one or more covariates, and compared to ANOVA, it reduces the variance for the estimators of interest. Recently, Wu and Ying [6] proposed the use of the empirical likelihood (EL) method to perform covariate adjustment for randomized clinical trials, as a nonparametric alternative to ANCOVA. This method allows the efficient incorporation of side information, such as the expected balance of the covariates between the treatment groups in a randomized study. Related nonparametric covariate adjustment methods can be developed by using the exponential tilting (ET) and continuous updated estimator (CUE) methods instead of the EL method.

In this paper, we evaluated the usefulness of three important members of the generalized empirical likelihood (GEL) family, including the EL, ET, and CUE methods, with respect to performing covariate adjustment for randomized studies in environmental research and public health. We have used these three methods because they are important members of the GEL family, and are implemented in the R package *gmm* [7,8]. Using a simulation study, we compared these three nonparametric covariate adjustment methods and ANCOVA. In addition to comparing ANCOVA with the three GEL methods, the paper also compared the three GEL methods among themselves, to identify if there is one among them that performs best in a consistent way. The evaluation of the performance of these four methods was based on the estimated root mean squared error (RMSE) and the empirical coverage for nominal 95% confidence intervals (CIs), for varying sample sizes, covariance structures, underlying distributions, and number of covariates, using 10,000 simulations per scenario.

## 2. Methods

### 2.1. Covariate Adjustment Methods

To compare outcome means between treatment groups, we use ANOVA (when we do not perform covariate adjustment) or ANCOVA (when we perform covariate adjustment), assuming that the error terms are independent, normally distributed, and with equal variance. For sensitivity analysis purposes, we may also want to use alternative statistical methods that do not make these parametric assumptions, to evaluate how robust the results of the ANOVA/ANCOVA methods are to their specific assumptions. In our paper, the covariate adjustment was performed using three GEL methods—EL estimation, ET estimation, and CUE—in addition to the ANCOVA method. The technical details regarding the GEL methods and the three nonparametric covariate adjustment methods based on the EL, ET, and CUE methods are included in the sections of the Appendix. Here, we are providing only a simplified description of these covariate adjustment methods to allow the reader to understand the main ideas underlying them.

For simplicity, let us consider a randomized study where we have two treatment groups—one outcome, and one covariate. We want to estimate the outcome mean difference between the two treatment groups with adjustment for the covariate. The GEL-based covariate adjustment methods start with all observations having uniform weights 1/n, where *n* is the total sample size. To estimate the outcome mean difference, we reweigh the observations as little as possible, as measured by a “distance” between the uniform weights 1/n and the new weights, such that the weighted means (using the new weights) for the covariate for the two treatment groups are equal (*i.e.*, covariate balance). The estimate of the outcome mean difference is the difference between the weighted means (using the new weights that provide covariate balance) for the outcome.

To construct the 95% confidence interval for the outcome mean difference by using the test inversion method, for each hypothesized value for the outcome mean difference, we reweigh the observations to achieve covariate balance and to have the outcome (weighted) mean difference equals the hypothesized value. If the new weights are “too far” from the uniform weights, we do not include that specific hypothesized value (for the outcome mean difference) in the 95% confidence interval. Conceptually, to construct the 95% confidence interval, we perform this for all possible values for the outcome mean difference. It is important to note that the only difference between the three GEL-based covariate adjustment methods is the specific measure used to quantify the “distance” between the uniform weights and the new weights.

### 2.2. Simulation Study

The simulation study had two goals. The first goal was to estimate the root mean squared error (RMSE) for each method using 10,000 simulations for each scenario. The second goal was to evaluate how well the nominal 95% CIs for the difference between means constructed by these methods cover the true mean difference (0, in our simulation study) by calculating the empirical coverage based on 10,000 simulations. The point estimates and corresponding 95% confidence intervals for the difference between means using the EL, ET, and CUE methods were constructed using the R package *gmm* [7,8]. These confidence intervals for the GEL methods that are constructed based on test inversion are only available starting with version 1.6 of the R package *gmm*.

Our simulation study is divided into three parts. In the first part, we consider situations involving equal sample sizes for the treatment groups, homoscedasticity, and no interaction between covariates and the treatment group. In the second part, we consider situations involving unequal sample sizes for the treatment groups, heteroscedasticity, and/or interactions between covariates and the treatment group. For both the first and the second part of the simulation study, we consider only the case when the true outcome mean difference is zero. In the third part of the simulation study, we use real data from Lanphear *et al.* [4] to investigate situations involving equal sample sizes for the treatment groups, homoscedasticity, and no interaction between covariates and the treatment group, similar to the first part of the simulation study, while considering situations where the true outcome mean difference is different from zero. We note that our simulation study is comprehensive by covering a broad range of possible situations and also by including simulations based on real data.

The general setup for the simulation study was as follows:

1. We estimated the difference between means and constructed corresponding 95% CIs, without adjustment and with adjustment for one covariate or two covariates;
2. We performed 10,000 simulations for each scenario under investigation;
3. We considered a sample size of 200 from which 200(1-δ) are assigned to group 1 (*z* = 0) and 200δ are assigned to group 2 (*z* = 1), where *δ* is between 0 and 1. Without loss of generality, the vector *z* is generated by setting the first 200(1-δ) elements to 0 and the remaining ones to 1;
4. For the underlying distributions of the data, we considered the following three types of multivariate distributions for $\left(y,x_{1},x_{2}\right)$, where *y* is the outcome and $x_{1}$ and $x_{2}$ are the covariates:
  (a) Normal (generated using the R package *mvtnorm* [9]);
  (b) t with three degrees of freedom (generated using the R package *mnormt* [10]);
  (c) Centered lognormal (generated using the R package *mvtnorm* [9]).
  For each distribution, $Var\left(y\right)=Var\left(x_{1}\right)=Var\left(x_{2}\right)=1$, $Cor\left(y,x_{1}\right)=Cor\left(y,x_{2}\right)=Cor\left(x_{1},x_{2}\right)=\rho$, and the three variables have mean 0. For the lognormal, which is the exponential of a multivariate normal with mean 0 and covariance matrix Σ, the multivariate normal was selected as to obtain the desired variances and correlations. We also subtracted from each variable its expected value.
5. In the simulation, we want to evaluate different scenarios. In particular, we want to allow for unequal assignment to the treatment groups, Var(y|z=1)≠Var(y|z=0), and/or $Cor\left(y,x_{i}|z=0\right)\neq Cor\left(y,x_{i}|z=1\right)$. In order to accomplish that, after generating the 200 observations, the outcome is modified as follows: Every $y_{i}$ with $z_{i}=1$ is multiplied by $v_{1}$, and then $\beta_{2}\left(x_{1i}+x_{2i}\right)$ is added, where $v_{1}$ is a parameter that affects the variance of *y* when z=1, and $\beta_{2}$ is another parameter that affects the correlation between *y* and the covariates when z=1. This modification has no effect on *y* when z=0, but it affects the variance of *y* and its correlation with the covariates when z=1 in the following way:
  $$Var\left(y|z=1\right)=v_{1}+2\rho k\beta_{2}\sqrt{v_{1}}+\beta_{2}^{2}k\left(1+\left(k-1\right)\rho \right),$$
  $$Cor\left(y,x_{j}|z=1\right)=\frac{\rho \sqrt{v_{1}}+\beta_{2}\left(1+\left(k-1\right)\rho \right)}{\sqrt{Var(y|z=1)}}.$$

#### 2.2.1. Equal Sample Sizes, Homoscedasticity, and No Interaction

For the first part of our simulation, we set δ=0.5, $v_{1}=1$, and $\beta_{2}=0$, which implies Var(y|z)=1 and $Cor(y,x_{i}|z)=\rho$ for the two treatment groups. In this set of simulations, we want to compare the properties of the four methods for different values of the correlation coefficient *ρ*. In particular, we consider *ρ* being equal to one of the following values: {0,0.1,0.3,0.5,0.7,0.9}.

We note that the simulated data satisfies the moment conditions for the GEL methods for all three distributions considered. The data simulated using the normal distribution satisfies the ANOVA/ANCOVA assumptions. The data simulated using the t distribution with three degrees of freedom and the lognormal distribution satisfies the ANOVA/ANCOVA assumptions except the normality assumption for the error terms, although the use of treatment groups with equal sample sizes makes the ANOVA/ANCOVA method robust to violations of the normality assumption, see [5] and [11]. Because of the randomization, there is no confounding due to the covariates. We are adjusting for covariates only to increase the efficiency of our estimators for the outcome mean difference between the two treatment groups.

#### 2.2.2. Unequal Sample Sizes, Heteroscedasticity, and/or Interaction

For the second part of the simulation study, we consider scenarios involving unbalanced treatment groups, heteroscedasticity, and/or interactions between covariates and treatment group. For each distribution, we consider five different combinations of the parameters $\left\{\delta ,v_{1},\beta_{2}\right\}$: Case 1: {0.2,2,0.5}, Case 2: {0.5,1,0.5}, Case 3: {0.2,1,0}, Case 4: {0.5,2,0}, and Case 5: {0.2,2,0}. The correlation coefficient *ρ* is set to 0.5 for all these five cases.

Specifically, Case 1 involves unequal group sizes, heteroscedasticity and interaction (Var(y|z=1)=4.16 and $Cor(y,x_{i}|z=1)=0.71$), Case 2 involves equal group sizes, homoscedasticity and interaction (Var(y|z=1)=2.75 and $Cor(y,x_{i}|z=1)=0.75$), Case 3 involves unequal group sizes, homoscedasticity and no interaction (Var(y|z=1)=1 and $Cor(y,x_{i}|z=1)=0.5$), Case 4 involves equal group sizes, heteroscedasticity and no interaction (Var(y|z=1)=2 and $Cor(y,x_{i}|z=1)=0.5$), and Case 5 involves unequal group sizes, heteroscedasticity and no interaction (Var(y|z=1)=2 and $Cor(y,x_{i}|z=1)=0.5$). We note that the validity of the GEL moment conditions is not affected by these changes, while the validity of the ANCOVA assumptions (*i.e.*, homoscedasticity, no covariate by treatment interaction) is affected.

#### 2.2.3. Real Data and Non-Null Effect Sizes

To enhance the paper, we have used real data from the randomized controlled trial described in Lanphear *et al.* [4] to perform additional simulations that are close to a real life situation, and also to illustrate the use of the four covariate adjustment methods with real data. We have used for this paper the data for the 169 children for whom both six-months baseline and 48-months follow-up blood lead concentrations are available. This includes 89 children randomized to the intervention group (group 2 or z=1, using the above terminology) and 80 children randomized to the control group (group 1 or z=0). Similar to the original study, we have used the natural log transformed blood lead concentration values instead of the original blood lead concentration values. The outcome for this experimental study was the natural log transformed blood lead concentration at the 48-month follow-up, while the covariate was the natural log transformed blood lead concentration at the six-months baseline.

For the third part of the simulation study, we have used descriptive statistics (means, standard deviations, and correlation coefficient) from the real data, to consider scenarios where the mean difference is not null, to allow us to compare the statistical power of the four different methods. We have expressed the mean difference in standard deviation units. The setup of the simulations was as follows: n=100 for each treatment group, {y,x} is a bivariate normal with mean {1.82+D(z),1.07} and covariance matrix Σ, with $\Sigma_{11}=0.58^{2}$, $\Sigma_{22}=0.52^{2}$, and $\Sigma_{12}=\Sigma_{21}=\left(0.35\right)\left(0.58\right)\left(0.52\right)$, where $\Sigma_{ij}$ is the element of Σ on the ith row and jth column, and the number of replications equals to 10,000. For the third part of the simulation study, we have δ=0.5, $v_{1}=1$, and $\beta_{2}=0$, which implies the same variance and correlation for the two groups. The correlation between *x* and *y* is therefore equal to 0.35. Here D(z)=Δ when z=0 (*i.e.*, the control group or group 1) and D(z)=0 when z=1 (*i.e.*, the intervention group or group 2), where Δ={0,0.1,0.2,0.3,0.4,0.5,0.6,0.7,0.8}. For Δ=0, we evaluate the size of the statistical tests, while, for all other values, we estimate the statistical power. We have simulated normal data because the distributions of the natural log transformed blood lead concentrations for the two treatment groups were approximately normal.

## 3. Results

Table 1, Table 2 and Table 3 show the estimated RMSE and the empirical coverage of nominal 95% confidence intervals for each one of the four covariate adjustment methods, separately for each scenario from the first part of the simulation study with a specified correlation structure and underlying distribution, based on 10,000 simulations. For completeness purposes, we are presenting the results without covariate adjustment, with adjustment for one covariate, and with adjustment for two covariates. It is important to note that even if baseline covariates are available, given the randomization, it is not required to adjust for the covariates.

Table 1 presents the simulation results for the situation involving no covariate adjustment (*i.e.*, either no covariate information is available, or the covariates are not used for adjustment although they are available). Overall, ANOVA and CUE perform equally well with respect to empirical coverage, and better than the EL and ET methods. The estimated RMSE for the EL method is smaller for the t-distribution and the lognormal distribution cases but that is associated with empirical coverage much below the nominal level.

Table 2 presents the simulation results for the situation involving adjustment for one covariate. For the normal distribution case, the estimated RMSE and empirical coverage are similar for the four methods. For the t-distribution case, CUE and ANCOVA perform equally well and better than the EL and ET methods, while having smaller estimated RMSE. For the lognormal distribution case, the performance of the EL and ET methods with respect to empirical coverage is even worst, while the estimated RMSE for CUE and ANCOVA tend to be smaller. We note that for completeness we have included the case when we adjust for a covariate that is uncorrelated with the outcome, although this situation is more of theoretical than practical interest. For each distribution under consideration, the results are consistent across the different correlation values. Similar conclusions apply to the simulation results from Table 3, where we adjust for two covariates that are correlated with the outcome and among themselves with the same correlation *ρ*.

Table 4, Table 5 and Table 6 present the results for the five different cases and the three distributions. Overall, CUE is the best method in terms of empirical coverage. ANCOVA performs poorly in Case 1 and Case 5, which are characterized by a high variance of the response variable for the smaller treatment group. This result indicates that CUE is robust to heteroscedasticity, while ANCOVA is not. However, we can see from the results from Table 7 that using the robust standard errors makes ANCOVA comparable to CUE in terms of empirical coverage.

The results from Table 8 indicate that CUE and ANCOVA provided the best control of the type I error, which corresponds to Δ=0, while having similar statistical power. It is important to note that the patterns of the estimated RMSE and empirical coverage results for these scenarios involving non-null mean differences were similar to those from the previous set of simulations that involved only null mean differences.

The results of the statistical analysis of the real data are presented in Table 9. The results of the four different covariate adjustment methods were similar for the parameter of main interest Δ, *i.e.*, the mean difference between the control group and the intervention group with respect to the natural log transformed blood lead concentration at the follow-up, adjusted for the natural log transformed blood lead concentration at the baseline. Given the very small estimates for Δ, we have provided all the results with five decimal places. Although, in the original study [4], there was no adjustment for the natural log transformed blood lead concentration at the baseline, these covariate adjusted results provide additional support for the conclusion that there was no significant effect of the intervention on the blood lead concentration. The results of the four methods were also similar for $\mu_{1}$, *i.e.*, the mean of the natural log transformed blood lead concentration at the follow-up for the intervention group, and for $\mu_{x}$, *i.e.*, the common mean of the natural log transformed blood lead concentration at the baseline. The table also illustrates the difference between the types of results provided by ANCOVA *versus* the GEL-based methods: the GEL methods provide an estimate for the common covariate mean ($\mu_{x}$), while ANCOVA provides an estimate for the slope for the linear relationship between the covariate and the outcome ($\beta_{x}$).

## 4. Conclusions

For our simulation study, we performed 10,000 simulations at different levels of treatment group sample size, covariance structure, underlying distribution, and number of covariates. We have also considered cases in which the variance of the outcome and its correlations with the covariates were different for the two treatment groups. We compared a parametric method, ANOVA/ANCOVA, and three GEL methods: the EL method, the ET method, and the CUE method. The main difference between ANCOVA and the GEL methods is that the former imposes an arbitrary parametric structure, while the latter methods only assume treatment randomization.

The results of the simulation study showed that, overall, the CUE-based covariate adjustment method and ANCOVA (with robust standard errors when heteroscedasticity is present) performed equally well and better than the covariate adjustment methods based on EL and ET. In terms of computational complexity, however, ANCOVA is clearly the simpler method since it relies on the least squares estimation method. Among the GEL methods considered here, EL is the least computationally stable, especially when the distribution of the variables has heavy tails. For example, for our scenarios involving the t-distribution, 20 to 30 simulations out of the total of 10,000 simulations per scenario involved lack of convergence. We should note that the results for the EL method may be improved by using the Bartlett correction or bootstrap calibration [14]. We have not investigated the usefulness of those two approaches in the current paper due to the additional computational complexity involved. In future research, we will consider alternative methods based on GEL which are less sensitive to distributions with heavy tails [15] and more computationally stable [16]. In addition, exploring other forms of heteroscedasticity and considering a more general set of moment conditions $E[z\left(f\left(x_{i}\right)-E\left(f\left(x_{i}\right)\right)\right]$ for general functions f() may help us identify situations for which the benefits of using GEL outweigh the computational complexity.

Based on the results of our simulation study, for sensitivity analysis purposes, we recommend the use of ANCOVA (with robust standard errors when heteroscedasticity is present) together with the CUE-based covariate adjustment method. This recommendation is based on the similar overall good performance in our simulation study of these two different statistical methods. If the results of the ANCOVA and the CUE-based covariate adjustment method imply similar conclusions, then the robustness of these conclusions is supported. If the results of these two different methods imply qualitatively different conclusions, then the conclusion implied by the CUE-based covariate adjustment method may be preferred given that this method only assumes that the treatment has been randomly assigned.

## Figures and Tables

**Table 1:** Estimated root mean squared error and empirical coverage of nominal 95% confidence intervals (no covariates).

	Normal		t With 3 df		Lognormal	
Method	RMSE	Coverage	RMSE	Coverage	RMSE	Coverage
EL	0.140678	0.9490	0.233912	0.9233	0.098365	0.9470
ET	0.140678	0.9484	0.245093	0.9419	0.098386	0.9473
CUE	0.140677	0.9502	0.245091	0.9512	0.098385	0.9520
ANOVA	0.140669	0.9507	0.245080	0.9516	0.098383	0.9520

EL: Empirical Likelihood, ET: Exponential Tilting, CUE: Continuous Updated Estimator, RMSE: Root Means Squared Error. The covariates were generated but they were not used for adjustment. Equal sample sizes, homoscedasticity, and no interaction. The table presents the case ρ=0.5. The results for the other values of *ρ* are identical.

**Table 2:** Estimated root mean squared error and empirical coverage of nominal 95% confidence intervals (1 covariate).

		Normal		t With 3 df		Lognormal	
Correlation	Method	RMSE	Coverage	RMSE	Coverage	RMSE	Coverage
ρ=0	EL	0.141107	0.9487	0.240249	0.9361	0.098066	0.9503
	ET	0.141102	0.9482	0.238359	0.9387	0.098050	0.9503
	CUE	0.141109	0.9496	0.236648	0.9503	0.098044	0.9544
	ANCOVA	0.141110	0.9499	0.236642	0.9501	0.098044	0.9544
ρ=0.1	EL	0.140015	0.9479	0.238037	0.9343	0.097411	0.9496
	ET	0.140010	0.9469	0.239149	0.9396	0.097386	0.9495
	CUE	0.140019	0.9499	0.237294	0.9514	0.097350	0.9537
	ANCOVA	0.140019	0.9498	0.237293	0.9515	0.097351	0.9535
ρ=0.3	EL	0.133528	0.9485	0.226304	0.9347	0.093231	0.9488
	ET	0.133528	0.9471	0.226028	0.9420	0.093092	0.9496
	CUE	0.133542	0.9505	0.224020	0.9539	0.092962	0.9552
	ANCOVA	0.133538	0.9508	0.224018	0.9540	0.092966	0.9552
ρ=0.5	EL	0.120711	0.9512	0.205436	0.9391	0.084588	0.9518
	ET	0.120718	0.9508	0.204373	0.9430	0.084314	0.9518
	CUE	0.120727	0.9527	0.202564	0.9538	0.084066	0.9580
	ANCOVA	0.120727	0.9530	0.202561	0.9544	0.084072	0.9579
ρ=0.7	EL	0.099358	0.9506	0.168965	0.9408	0.069797	0.9513
	ET	0.099364	0.9498	0.167957	0.9423	0.069404	0.9519
	CUE	0.099374	0.9518	0.166543	0.9531	0.069065	0.9582
	ANCOVA	0.099374	0.9520	0.166538	0.9533	0.069072	0.9581
ρ=0.9	EL	0.060800	0.9522	0.105895	0.9405	0.042669	0.9521
	ET	0.060802	0.9513	0.103060	0.9445	0.042314	0.9533
	CUE	0.060809	0.9539	0.102212	0.9562	0.042018	0.9598
	ANCOVA	0.060808	0.9540	0.102210	0.9559	0.042024	0.9599

EL: Empirical Likelihood, ET: Exponential Tilting, CUE: Continuous Updated Estimator, RMSE: Root Means Squared Error. Equal sample sizes, homoscedasticity, and no interaction.

**Table 3:** Estimated root mean squared error and empirical coverage of nominal 95% confidence intervals (2 covariates).

		Normal		t With 3 df		Lognormal	
Correlation	Method	RMSE	Coverage	RMSE	Coverage	RMSE	Coverage
ρ=0	EL	0.141501	0.9488	0.239127	0.9334	0.098215	0.9497
	ET	0.141474	0.9477	0.234743	0.9377	0.098202	0.9491
	CUE	0.141472	0.9504	0.231773	0.9516	0.098202	0.9531
	ANCOVA	0.141474	0.9504	0.231768	0.9516	0.098201	0.9532
ρ=0.1	EL	0.139807	0.9484	0.236093	0.9344	0.097183	0.9494
	ET	0.139776	0.9471	0.232302	0.9397	0.097117	0.9502
	CUE	0.139771	0.9510	0.229410	0.9529	0.097053	0.9539
	ANCOVA	0.139773	0.9514	0.229407	0.9532	0.097055	0.9538
ρ=0.3	EL	0.130232	0.9503	0.220592	0.9367	0.090962	0.9498
	ET	0.130210	0.9495	0.216977	0.9405	0.090671	0.9500
	CUE	0.130207	0.9534	0.214185	0.9544	0.090447	0.9551
	ANCOVA	0.130207	0.9536	0.214181	0.9543	0.090454	0.9552
ρ=0.5	EL	0.114121	0.9512	0.193992	0.9352	0.080025	0.9507
	ET	0.114112	0.9500	0.190474	0.9387	0.079533	0.9504
	CUE	0.114119	0.9531	0.188074	0.9529	0.079165	0.9561
	ANCOVA	0.114118	0.9535	0.188062	0.9528	0.079176	0.9562
ρ=0.7	EL	0.090880	0.9513	0.154551	0.9353	0.063826	0.9504
	ET	0.090881	0.9506	0.151771	0.9396	0.063243	0.9516
	CUE	0.090895	0.9534	0.149871	0.9521	0.062805	0.9573
	ANCOVA	0.090896	0.9536	0.149861	0.9516	0.062816	0.9572
ρ=0.9	EL	0.053779	0.9507	0.094089	0.9362	0.037700	0.9487
	ET	0.053795	0.9498	0.089999	0.9401	0.037238	0.9509
	CUE	0.053807	0.9531	0.088868	0.9542	0.036892	0.9579
	ANCOVA	0.053807	0.9534	0.088863	0.9541	0.036900	0.9580

EL: Empirical Likelihood, ET: Exponential Tilting, CUE: Continuous Updated Estimator, RMSE: Root Means Squared Error. Equal sample sizes, homoscedasticity, and no interaction.

**Table 4:** Estimated root mean squared error and empirical coverage of nominal 95% confidence intervals (no covariates).

		Normal		t With 3 df		Lognormal	
	Method	RMSE	Coverage	RMSE	Coverage	RMSE	Coverage
Case 1	EL	0.33412	0.9419	0.54806	0.9301	0.23527	0.9420
	ET	0.33411	0.9415	0.60446	0.9341	0.23543	0.9407
	CUE	0.33414	0.9495	0.60463	0.9563	0.23541	0.9432
	ANOVA	0.33418	0.8129	0.60464	0.8196	0.23537	0.8177
Case 2	EL	0.19313	0.9496	0.32942	0.9430	0.13611	0.9449
	ET	0.19313	0.9484	0.33507	0.9441	0.13611	0.9435
	CUE	0.19313	0.9511	0.33508	0.9525	0.13611	0.9480
	ANOVA	0.19314	0.9509	0.33508	0.9525	0.13611	0.9478
Case 3	EL	0.17703	0.9440	0.29451	0.9335	0.12452	0.9444
	ET	0.17703	0.9438	0.32076	0.9377	0.12466	0.9439
	CUE	0.17703	0.9500	0.32077	0.9537	0.12466	0.9476
	ANOVA	0.17704	0.9498	0.32076	0.9481	0.12466	0.9542
Case 4	EL	0.17195	0.9494	0.29431	0.9426	0.12121	0.9482
	ET	0.17195	0.9489	0.30069	0.9441	0.12121	0.9479
	CUE	0.17195	0.9499	0.30070	0.9530	0.12121	0.9508
	ANOVA	0.17195	0.9499	0.30070	0.9529	0.12121	0.9506
Case 5	EL	0.23777	0.9430	0.39245	0.9314	0.16759	0.9412
	ET	0.23777	0.9426	0.43216	0.9350	0.16775	0.9411
	CUE	0.23778	0.9499	0.43217	0.9564	0.16775	0.9432
	ANOVA	0.23776	0.8873	0.43218	0.8906	0.16774	0.8947

EL: Empirical Likelihood, ET: Exponential Tilting, CUE: Continuous Updated Estimator, RMSE: Root Means Squared Error. Case 1: unequal group sizes, heteroscedasticity and interaction. Case 2: equal group sizes, homoscedasticity and interaction. Case 3: unequal group sizes, homoscedasticity and no interaction. Case 4: equal group sizes, heteroscedasticity and no interaction. Case 5: unequal group sizes, heteroscedasticity and no interaction.

**Table 5:** Estimated root mean squared error and empirical coverage of nominal 95% confidence intervals (1 covariate).

		Normal		t With 3 df		Lognormal	
	Method	RMSE	Coverage	RMSE	Coverage	RMSE	Coverage
Case 1	EL	0.24948	0.9389	0.41290	0.9209	0.17494	0.9343
	ET	0.24918	0.9388	0.41240	0.9290	0.17425	0.9338
	CUE	0.24914	0.9502	0.40749	0.9524	0.17381	0.9404
	ANCOVA	0.26775	0.8327	0.44075	0.8428	0.18674	0.8369
Case 2	EL	0.14666	0.9525	0.24940	0.9402	0.10328	0.9507
	ET	0.14665	0.9511	0.24716	0.9426	0.10296	0.9495
	CUE	0.14666	0.9532	0.24477	0.9538	0.10287	0.9521
	ANCOVA	0.14664	0.9533	0.24476	0.9535	0.10286	0.9523
Case 3	EL	0.15474	0.9409	0.25719	0.9216	0.10854	0.9372
	ET	0.15454	0.9403	0.25697	0.9284	0.10820	0.9388
	CUE	0.15450	0.9494	0.25429	0.9516	0.10796	0.9459
	ANCOVA	0.15362	0.9492	0.25781	0.9482	0.10726	0.9504
Case 4	EL	0.14832	0.9533	0.25233	0.9423	0.10431	0.9525
	ET	0.14833	0.9523	0.25135	0.9442	0.10391	0.9525
	CUE	0.14834	0.9546	0.24918	0.9556	0.10362	0.9567
	ANCOVA	0.14834	0.9545	0.24917	0.9558	0.10363	0.9564
Case 5	EL	0.20857	0.9407	0.34501	0.9198	0.14655	0.9358
	ET	0.20828	0.9402	0.34472	0.9276	0.14609	0.9365
	CUE	0.20824	0.9505	0.34096	0.9504	0.14575	0.9419
	ANCOVA	0.20781	0.8886	0.34659	0.8935	0.14541	0.8925

EL: Empirical Likelihood, ET: Exponential Tilting, CUE: Continuous Updated Estimator, RMSE: Root Means Squared Error. Case 1: unequal group sizes, heteroscedasticity and interaction. Case 2: equal group sizes, homoscedasticity and interaction. Case 3: unequal group sizes, homoscedasticity and no interaction. Case 4: equal group sizes, heteroscedasticity and no interaction. Case 5: unequal group sizes, heteroscedasticity and no interaction.

**Table 6:** Estimated root mean squared error and empirical coverage of nominal 95% confidence intervals (2 covariates).

		Normal		t With 3 df		Lognormal	
	Method	RMSE	Coverage	RMSE	Coverage	RMSE	Coverage
Case 1	EL	0.213311	0.9374	0.351761	0.9182	0.150503	0.9296
	ET	0.212692	0.9370	0.341089	0.9276	0.149500	0.9291
	CUE	0.212640	0.9505	0.336064	0.9520	0.149057	0.9363
	ANCOVA	0.240902	0.8487	0.383987	0.8592	0.167813	0.8527
Case 2	EL	0.129098	0.9517	0.217790	0.9365	0.091147	0.9487
	ET	0.129077	0.9509	0.213566	0.9410	0.090744	0.9482
	CUE	0.129083	0.9546	0.210669	0.9564	0.090791	0.9497
	ANCOVA	0.129070	0.9540	0.210647	0.9554	0.090769	0.9496
Case 3	EL	0.147092	0.9391	0.242755	0.9162	0.103535	0.9323
	ET	0.146641	0.9403	0.236627	0.9251	0.102864	0.9350
	CUE	0.146557	0.9524	0.233094	0.9507	0.102515	0.9443
	ANCOVA	0.144683	0.9505	0.237339	0.9477	0.101013	0.9536
Case 4	EL	0.140630	0.9510	0.238623	0.9363	0.098951	0.9508
	ET	0.140621	0.9502	0.234311	0.9401	0.098341	0.9515
	CUE	0.140628	0.9529	0.231323	0.9534	0.097910	0.9562
	ANCOVA	0.140627	0.9532	0.231313	0.9531	0.097920	0.9556
Case 5	EL	0.198637	0.9378	0.325978	0.9128	0.140084	0.9306
	ET	0.197985	0.9377	0.317462	0.9224	0.139167	0.9314
	CUE	0.197898	0.9521	0.312614	0.9533	0.138652	0.9396
	ANCOVA	0.196377	0.8919	0.318986	0.8951	0.137360	0.8901

EL: Empirical Likelihood, ET: Exponential Tilting, CUE: Continuous Updated Estimator, RMSE: Root Means Squared Error. Case 1: unequal group sizes, heteroscedasticity and interaction. Case 2: equal group sizes, homoscedasticity and interaction. Case 3: unequal group sizes, homoscedasticity and no interaction. Case 4: equal group sizes, heteroscedasticity and no interaction. Case 5: unequal group sizes, heteroscedasticity and no interaction.

**Table 7:** Estimated empirical coverage of nominal 95% confidence intervals for ANCOVA with robust standard errors.

	Covariates	Normal	t With 3 df	Lognormal
Case 1	0	0.9451	0.9514	0.9408
	1	0.9490	0.9515	0.9380
	2	0.9479	0.9523	0.9374
Case 2	0	0.9505	0.9525	0.9476
	1	0.9542	0.9583	0.9544
	2	0.9552	0.9618	0.9519
Case 3	0	0.9470	0.9520	0.9469
	1	0.9467	0.9540	0.9494
	2	0.9519	0.9576	0.9510
Case 4	0	0.9498	0.9527	0.9505
	1	0.9545	0.9580	0.9573
	2	0.9546	0.9587	0.9563
Case 5	0	0.9469	0.9532	0.9413
	1	0.9463	0.9525	0.9427
	2	0.9491	0.9560	0.9425

The robust standard errors are computed using the HC3 type of heteroscedasticity consistent covariance matrices [12,13]. Case 1: unequal group sizes, heteroscedasticity and interaction. Case 2: equal group sizes, homoscedasticity and interaction. Case 3: unequal group sizes, homoscedasticity and no interaction. Case 4: equal group sizes, heteroscedasticity and no interaction. Case 5: unequal group sizes, heteroscedasticity and no interaction.

**Table 8:** Statistical power for ANCOVA and the GEL-based covariate adjustment methods.

Method	Δ=0	Δ=0.1	Δ=0.2	Δ=0.3	Δ=0.4	Δ=0.5	Δ=0.6	Δ=0.7	Δ=0.8
EL	0.0515	0.1216	0.3287	0.6180	0.8512	0.9653	0.9944	0.9996	1.0000
ET	0.0518	0.1227	0.3305	0.6200	0.8520	0.9659	0.9945	0.9996	1.0000
CUE	0.0499	0.1186	0.3229	0.6113	0.8474	0.9638	0.9941	0.9996	1.0000
ANCOVA	0.0497	0.1174	0.3221	0.6101	0.8467	0.9636	0.9941	0.9996	1.0000

Equal sample sizes, homoscedasticity, and no interaction; The table provides the empirical type I error rates for Δ=0 that corresponds to the null hypothesis of equal means.

**Table 9:** Results of the statistical analysis of the data from the randomized study described in Lanphear *et al.* (2000) [4].

	EL	ET	CUE	ANCOVA
$\mu_{1}$	1.82324	1.82305	1.82289	1.82185
	(1.69919;1.95209)	(1.69821;1.95022)	(1.69369;1.95091)	(1.70175;1.94195)
Δ	0.00453	0.00450	0.00448	0.00431
	(-0.16024;0.16635)	(-0.15954;0.16720)	(-0.16109;0.17008)	(-0.16132;0.16993)
$\mu_{x}$	1.07772	1.07743	1.07715	
	(1.00186;1.15638)	(1.00076;1.15546)	(0.99789;1.15594)	
$\beta_{x}$				0.38249
				(0.22383;0.54116)

$\mu_{1}$: mean of the natural log transformed blood lead concentration at the follow-up for the intervention group; Δ: mean difference between the groups with respect to the natural log transformed blood lead concentration at the follow-up; $\mu_{x}$: common mean of the natural log transformed blood lead concentration at the baseline; $\beta_{x}$: slope for the linear relationship between the natural log transformed blood lead concentration at the baseline and the natural log transformed blood lead concentration at the follow-up.

## Appendix A. Minimum Discrepancy Methods

Cressie and Read [17] have unified the goodness-of-fit statistics for multinomial data using their power-divergence family of statistics. Estimation methods for discrete multivariate data based on the Cressie and Read (C–R) power-divergence family involve the constrained minimization of a divergence measure between the observed and the expected probability distributions. This class of estimation methods includes many important special cases, such as the empirical likelihood (EL) estimation, exponential tilting (ET) estimation, and the continuous updated estimation (CUE) [14]. Newey and Smith [18] describe the related generalized empirical likelihood (GEL) family.

Before presenting the GEL methods, we first provide a brief introduction to the minimum discrepancy (MD) methods. In particular, we are focusing on the MD methods based on the C–R family, because the GEL methods are closely related to them. The C–R power-divergence family of statistics, proposed in [19] and unified in [17] by Cressie and Read for multinomial data, can be written as (Note that we use the parametrization from Newey and Smith [18].)

(A1) $$CR\left(\pi ;a\right)=\frac{1}{a(a+1)}\sum_{i=1}^{n}\left[{\left(n\pi_{i}\right)}^{a+1}-1\right]/n,$$

where *a* is a parameter that identifies the specific member of the family, and for a=0 and a=-1 the function is defined as the limit. As noted by Owen [14], the limiting cases CR(π;-1) and CR(π;0), correspond to the EL method and the ET method, respectively. Furthermore, the value a=1 corresponds to Neyman’s minimum chi-square method or the Euclidean empirical likelihood method [14,20].

This CR(π;a) function measures the discrepancy between the probability distribution *π* and the uniform probability distribution that assigns a probability 1/n to each observation. We can easily verify that CR(1/n;a)=0, and also that it is strictly positive for any other probability distribution *π*, for all *a*. In many applications, we are interested in estimating an unknown parameter vector *θ*, using a set of estimating equations that we know or assume to be valid. In most cases, these estimating equations can be expressed as a vector of moment conditions E[g(x;θ)]=0. When the number of moment conditions is equal to the dimension of the parameter vector, *θ* can be estimated simply by solving the system of possibly nonlinear equations $\sum_{i=1}^{n}\left(1/n\right)g\left(x_{i};\theta \right)=0$. In other words, we replace the population moment conditions by their sample versions, using the uniform probability distribution as an estimate of the true probability distribution. On the other hand, if we have more conditions than the number of parameters, there is no solution to the system of estimating equations. However, under some regularity conditions, there exist infinitely many sets of non-negative probabilities $\left\{\pi_{1},...,\pi_{n}\right\}$ satisfying the condition $\sum_{i=1}^{n}\pi_{i}=1$, for which the system of equations $\sum_{i=1}^{n}\pi_{i}g\left(x_{i};\theta \right)=0$ has a solution. The MD methods involve finding a set of probabilities $\left\{{\hat{\pi }}_{1},...,{\hat{\pi }}_{n}\right\}$ and an estimate $\hat{\theta }$, by minimizing CR(π;a) subject to the constraint $\sum_{i=1}^{n}\pi_{i}g\left(x_{i};\theta \right)=0$ for a fixed *a*. The objective is therefore to be as close as possible to the uniform probability distribution, which is the best nonparametric estimate of the true distribution, while satisfying the moment conditions. Of course, our estimator $\hat{\theta }$ depends on the value of *a* which specifies the discrepancy.

## Appendix B. Generalized Empirical Likelihood Methods

All MD estimators based on CR(π;a) belong to the family of GEL estimators [18] (More specifically, the estimator $\hat{\theta }$ is the solution to $\min_{\theta }\left\{\max_{\lambda }\sum_{i=1}^{n}\left[\rho \left(\lambda^{\prime }g\left(x_{i},\theta \right)\right)-\rho \left(0\right)\right]\right\}$, where the function ρ(v) depends on *a*. It is the dual of the MD problem and its objective function is numerically identical to the CR function at the optimum.). In particular, Smith [21] shows that this is the case for the EL and ET methods, while Newey and Smith [18] show that the Euclidean empirical likelihood estimator [20] is the same as the CUE estimator [22]. The advantage of the GEL methods is that they offer a numerically more tractable way of solving the MD estimation problem, and they also make it easier to derive the theoretical properties of the resulting estimators. Because it is more common to refer to the GEL methods than to the MD methods when the estimating equations are based on moment conditions, the former term will be preferred for the rest of this paper.

Although all GEL estimators are asymptotically equivalent to the generalized method of moments (GMM) estimator [23], their small sample properties are different [18]. For example, Newey and Smith [18] show that in some cases the bias of the EL estimator converges to zero faster than for the other GEL methods. However, those results are only valid in large samples. Small sample properties can only be evaluated through simulation studies.

One advantage of the GEL methods over the ordinary least squares (OLS) method used by ANCOVA for covariate adjustment is that it allows us to incorporate more information through additional moment conditions. This is an important advantage since more information usually translates into higher statistical efficiency for the estimators. The GEL methods also allow us to test whether the moment conditions are valid. Suppose E[g(x;θ)]=0 represents our *q* moment conditions, and suppose *θ* is a parameter vector of dimension k<q. In this case the model is said to be over-identified. If the moment conditions are valid, $2nCR(\hat{\pi };a)$ is asymptotically distributed as a chi-squared distribution with (q-k) degrees of freedom [18]. For example, if we add conditions that are only valid when we have a randomized experiment, we could test whether the randomization has been properly performed.

We can also test the null hypothesis $H_{0}:\theta_{i}=c$, for one element of interest of *θ*, as follows. First, we fix $\theta_{i}$ to its value under the null hypothesis, and then we estimate the remaining elements of *θ* using GEL methods. Let $\tilde{R}\left(c\right)=2nCR\left(\tilde{\pi };a\right)$ be the solution under the null hypothesis, and $\hat{R}=2nCR\left(\hat{\pi };a\right)$ be the solution under the unrestricted model. Then, if the null hypothesis is true, $Q\left(c\right)=\tilde{R}\left(c\right)-\hat{R}$ is asymptotically distributed as a chi-squared distribution with 1 degree of freedom by a result similar to Wilk’s Theorem. Furthermore, we can construct a nonparametric 95% confidence interval for $\theta_{i}$ by inverting the statistical test, *i.e.*, by searching for all values of *c* that are such that Q(c)<3.8415, the critical value of the chi-squared distribution with 1 degree of freedom. This is how we have constructed our confidence intervals for the three GEL methods presented in this paper. This approach is computationally intensive because we need to estimate the restricted model for all values of *c* (*i.e.*, the implied probabilities ${\tilde{\pi }}_{i}$ are functions of *c*, and they must be recomputed each time) (This is how the function confint with the option type=’invLR’ constructs the confidence intervals in version 1.6 and above of the R package *gmm* [7].). However, this approach is more flexible than the OLS-based approach to construct confidence intervals because it does not require the confidence intervals to be symmetrical around the point estimate.

## Appendix C. Moment Conditions

In our study, we consider the estimation of the difference between two means without adjustment for covariates as well as with adjustment for one or two covariates. For the situation involving no covariate adjustment, the moment conditions can be written as follows:

(A2) $$E\left(\begin{array}{c} \left(y-\mu_{1}-\Delta z\right)\\ z\times (y-\mu_{1}-\Delta z) \end{array}\right)=0,$$

where y is the dependent variable, z is a binary treatment group indicator (z=0 for group 1, and *z* = 1 for group 2), $\mu_{1}$ is the mean of the outcome for group 1, and Δ is the difference between the means of the outcome for the two groups. The number of moment conditions is equal to the number of parameters $\left\{\mu_{1},\Delta \right\}$, which implies that the point estimates from any GEL methods will be identical to the OLS estimate, although the confidence intervals will be different as described above. To illustrate the procedure described in the previous section, if we want to test the hypothesis $H_{0}:\Delta =c$, we have to estimate $\mu_{1}$ after imposing this null hypothesis. The restricted model is therefore over-identified (k=1, q=2). The confidence interval is constructed by searching for all *c* that are such that Q(c)<3.8415, where $Q\left(c\right)=\tilde{R}\left(c\right)$ because $\hat{R}=0$ (${\hat{\pi }}_{i}=1/n$ for the just-identified models).

For the situation involving adjustment for one covariate, the moment conditions can be written as follows:

(A3) $$E\left(\begin{array}{c} \left(x-\mu_{x}\right)\\ z\times (x-\mu_{x})\\ y-\mu_{1}-\Delta z\\ z\times (y-\mu_{1}-\Delta z) \end{array}\right)=0,$$

where x is the covariate, $\mu_{x}$ is the common mean of the covariate for both groups, and the other notations are the same as for the case involving no adjustment. Here, we add two additional moment conditions for the covariate x to incorporate the information that we expect the two treatment groups to be balanced with respect to the covariate. We could add many more moment conditions because in randomized experiments E[z(f(x)-E(f(x)))]=0 for all functions *f*. In our paper, we only analyze the simplest case f(x)=x.

For the situation involving adjustment for two covariates, the moment conditions can be written as follows:

(A4) $$E\left(\begin{array}{c} \left(x_{1}-\mu_{x_{1}}\right)\\ z\times (x_{1}-\mu_{x_{1}})\\ \left(x_{2}-\mu_{x_{2}}\right)\\ z\times (x_{2}-\mu_{x_{2}})\\ \left(y-\mu_{1}-\Delta z\right)\\ z\times (y-\mu_{1}-\Delta z) \end{array}\right)=0,$$

where $x_{1}$ and $x_{2}$ are two covariates, $\mu_{x_{1}}$ and $\mu_{x_{2}}$ are the common means of the first and second covariate for both groups, respectively, and the other notations are the same as for the case involving no adjustment.
